# Supplementary material for: Deciphering temporal antifungal dynamics of a rare actinomycete via integrated omics
Source: mSystems. 2026 Sep 28;11(10):e00694-26. doi: 10.1128/msystems.00694-26 (PMC13712795; doi:10.1128/msystems.00694-26)
Supplement: Supplementary file 1 [file reviewer-comments.pdf]

ASM | mSystems

Deciphering temporal antifungal dynamics of a rare actinomycete via integrated omics

Eddie Cytryn, Michael Dubovis, Ivan Plyushchenko, Judith Kraut-Cohen, Tal Luzzatto-Knaan, Jonathan Friedman, and Hildah Amutuhaire

Corresponding Author(s): Eddie Cytryn, The Volcani Center, Agricultural Research Organization

Review Timeline:

Submission Date:	May 28, 2026
Editorial Decision:	July 1, 2026
Revision Received:	July 13, 2026
Editorial Decision:	August 14, 2026
Revision Received:	August 19, 2026
Accepted:	September 2, 2026

Editor: Trent Northen

Reviewer(s): Disclosure of reviewer identity is with reference to reviewer comments included in decision letter(s). The following individuals involved in review of your submission have agreed to reveal their identity: Justin J J van der Hooft (Reviewer #1)

Transaction Report:

(Note: With the exception of the correction of typographical or spelling errors that could be a source of ambiguity, letters and reports are not edited. The original formatting of letters and referee reports may not be reflected in this compilation.)

DOI: https://doi.org/10.1128/msystems.00694-26

Re: mSystems00694-26 (**Deciphering temporal antifungal dynamics of a rare actinomycete via integrated omics**)

Dear Dr. Eddie Cytryn:

Thank you for the privilege of reviewing your work. Below you will find my comments, instructions from the mSystems editorial office, and the reviewer comments.

Please return the manuscript within 60 days; if you cannot complete the modification within this time period, please contact me. If you do not wish to modify the manuscript and prefer to submit it to another journal, notify me immediately so that the manuscript may be formally withdrawn from consideration by mSystems.

Revision Guidelines
To submit your modified manuscript, log into the submission site at https://msystems.msubmit.net/cgi-bin/main.plex. Go to Author Tasks and click the appropriate manuscript title to begin. The information you entered when you first submitted the paper will be displayed; update this as necessary. Note the following requirements:

• Upload point-by-point responses to the issues raised by the reviewers in a file named "Response to Reviewers," NOT in your cover letter.
• Upload a compare copy of the manuscript (without figures) as a "Marked-Up Manuscript" file.
• Upload a clean .DOC/.DOCX version of the revised manuscript and remove the previous version.
• Each figure must be uploaded as a separate, editable, high-resolution file (TIFF or EPS preferred), and any multipanel figures must be assembled into one file.
• Any supplemental material intended for posting by ASM should be uploaded with their legends separate from the main manuscript. You can combine all supplemental material into one file (preferred) or split it into a maximum of 10 files with all associated legends included.

For complete guidelines on revision requirements, see our Submission and Review Process webpage. Submission of a paper that does not conform to guidelines may delay acceptance of your manuscript.

Data availability: ASM policy requires that data be available to the public upon online posting of the article, so please verify all links to sequence records, if present, and make sure that each number retrieves the full record of the data. If a new accession number is not linked or a link is broken, provide mSystems production staff with the correct URL for the record. If the accession numbers for new data are not publicly accessible before the expected online posting of the article, publication may be delayed; please contact production staff (mSystems@asmusa.org) immediately with the expected release date.

Publication Fees: For information on publication fees and which article types are subject to charges, visit our website. If your manuscript is accepted for publication and any fees apply, you will be contacted separately about payment during the production process; please follow the instructions in that e-mail. Arrangements for payment must be made before your article is published.

ASM Membership: Corresponding authors may join or renew ASM membership to obtain discounts on publication fees. Need to upgrade your membership level? Please contact Customer Service at Service@asmusa.org.

The ASM Journals program strives for constant improvement in our submission and publication process. Please tell us how we can improve your experience by taking this quick Author Survey.

Thank you for submitting your paper to mSystems.

Sincerely,
Trent Northen
Editor
mSystems

Reviewer #1 (Comments for the Author):

The revised manuscript by Amutuhaire and co-workers has improved in clarity over the previous version. I thank the authors for carefully considering the previous feedback, and applaud their thorough modifications to both reviewers' comments and suggestions. Having said that, there are several items that still need additional clarification:

Data availability: the authors have uploaded their metabolomics data into MassIVE, but the data is still "private" and no reviewer (and editor) acces has been provided. Please ensure that the data becomes public so it is visible and (re)usable. Furthermore, I appreciate that the authors followed the suggestion for the Zenodo repository, and also connected their genomes with the metabolomes in the Paired Omics Data Platform. Could the authors also include a reference to the Paired Omics Data Platform resource?

Chemically annotated features: the authors clarified (lines 516 - 528) were the chemical compound class predictions came from, but it is unclear where the now introduced "255 retained features came from. In lines 511-512, I could trace back the number, but it is unclear i) how many features were detected in total (using basic noise filtering), and ii) in which program/how the further filtering was done. Also, what do the authors mean with "single-sample features"?
Furthermore, could the authors specify the chemical classification system they used? I.e. the term "pathways" suggests to me that the authors used NPClassifier ontology? If indeed, can they make this explicit and also refer to the resource?

Scalable strategy: the authors claim in the discussion that their strategy is scalable:
"Thus, while this study does not establish causal BGC-metabolite relationships, it provides a scalable prioritization strategy for selecting BGCs, regulators, growth stages, and metabolite features for downstream functional validation."
However, I miss the arguments/proof. Also, if it is so scalable as this sentence implies, how come the authors did not use/produce more omics data? The point needs some additional work to ensure that the lessons learnt from the current study will and can be used by others: is the strategy scalable by design? What makes it scalable? How come relatively little omics data was used in the current study? (this is a key thing to highlight as a lower number also impacts the chance of finding relevant links across omics layers) What are the least scalable parts and any mitigations or ideas on how to improve/accelerate these parts? Given the focus of the work on the prioritization framework, I think this should be worked out more. I value some of the other insights now mentioned in the discussion and I very much welcome the authors to also extend on those.

General comment: the line numbers in the response letter did not always correspond with the actual line numbers in the manuscript. My recommendation would be to always add the added manuscript sections to the response letter, so that it takes less time to track them in the manuscript.

Reviewer #2 (Comments for the Author):

The revised manuscript addresses most of the concerns raised in the previous rounds. There are, nevertheless, several parts where descriptions are either ambiguous or not completely supported by the data. These need to be corrected prior to publication.

L29. I think what's unknown is their products, not the functions.

L91-94. The papers cited here primarily discuss or apply integrating genomics and metabolomics. Transcriptomics is little taken account of if at all. Please cite a few articles where transcriptomics and metabolomics are integratively analysed or its need is more in depth discussed.

L175. Convert the centrifugation unit from rpm to xg.

L217-218. Describe what "alignments were processed" means.

L262. Although the parameters are provided in Zenodo, please describe some key parameters here such as the ppm and m/z tolerances, minimum peak height and peak width range.

Figs 1C and 1E, and other similarly made figures. It is still unclear what each character means. Describe what each of them is in the figure legend. For example, what does "B" compare to in Fig 1C?

L361. Please provide the molar concentration of cycloheximide used.

L377-378. This sentence is ambiguous. Was a subset of differentially expressed genes that had functional annotations used? Or were all the differentially expressed genes with functional annotations used? If the former is correct, please provide a justification for using only the subset. If the latter is the case, please rephrase this sentence.

L416. Were z scores used for determining differentially expressed core biosynthetic genes instead of the fold changes? Please clarify which values were used and the criteria.

L419-420. TPM values themselves do not indicate whether a given is more highly transcribed than others. Rephrase this to accurately describe what is measured/compared.

L423-428. Please cite papers supporting these claims.

L443. BGCs do not encode metabolites. Rephrase it.

Fig. S4. "Non-significant genes" sound as if they are irrelevant. Rephrase it.

L483. It can be these regulators are irrelevant to the BGC expression. Since there's no evidence of multilayered architecture, state that this is a possibility, not the definitive mechanism.

L485. What is shown is expression patterns and the relatively large number of TFs within the BGC, not regulatory architecture. Rephrase it to accurately indicate what the data and information show.

L511. There is an unnecessary dot.

L577. Show quantities of this LC-MS feature over time from the time-series metabolomics data.

L635. Again, TPM values do not indicate whether genes are significantly expressed. Change the tone of this claim. Also, prior studies do not claim that transcriptional inactivity is a universal feature.

L650-656. Since there is no evidence that the analysed CSRs are indeed controlling expression of the BGCs, state that this is a possibility.

Reviewer #1 (Comments for the Author):

The revised manuscript by Amutuhaire and co-workers has improved in clarity over the previous version. I thank the authors for carefully considering the previous feedback, and applaud their thorough modifications to both reviewers' comments and suggestions. Having said that, there are several items that still need additional clarification:

Data availability: the authors have uploaded their metabolomics data into MassIVE, but the data is still "private" and no reviewer (and editor) access has been provided. Please ensure that the data becomes public so it is visible and (re)usable. Furthermore, I appreciate that the authors followed the suggestion for the Zenodo repository, and also connected their genomes with the metabolomes in the Paired Omics Data Platform. Could the authors also include a reference to the Paired Omics Data Platform resource?

The data in MassIVE has been made public and a reference to the Paired Omics Data Platform resource added on line 324.

Chemically annotated features: the authors clarified (lines 516 - 528) were the chemical compound class predictions came from, but it is unclear where the now introduced "255 retained features came from. In lines 511-512, I could trace back the number, but it is unclear i) how many features were detected in total (using basic noise filtering), and ii) in which program/how the further filtering was done. Also, what do the authors mean with "single-sample features"? Furthermore, could the authors specify the chemical classification system they used? I.e. the term "pathways" suggests to me that the authors used NPClassifier ontology? If indeed, can they make this explicit and also refer to the resource?

We revised the Methods and Results to clarify the feature-filtering workflow and the chemical classification system used. In the Methods section, lines 285-288, we added the following text:

"CANOPUS-based chemical classifications were interpreted using the NPClassifier ontology (46), which assigns natural products to pathway, superclass, and class levels; thus, 'pathway' refers to the NPClassifier chemical ontology level."

In the Methods section, lines 295-301, we further clarified the filtering procedure as follows:

"In MetaboAnalyst, blank subtraction was applied using the default sample/blank ratio threshold of 10, medium-associated features were removed based on medium controls, and features detected in only one biological sample were removed as non-reproducible singleton features. Low-variance filtering was performed using the default interquartile range criterion, removing the lowest 25% of features, while no low-abundance filtering was applied. The resulting filtered feature table was used for PCA and MaAsLin2 analysis."

In the Results section, lines 518 - 522, we clarified the origin of the 255 retained features as follows:

“Following peak detection, alignment, and feature quantification in MZmine, 943 metabolite features were detected across biological samples, blank QC injections, and medium controls. The aligned feature table was then filtered in MetaboAnalyst 6.0 to remove blank-associated signals, medium-derived features, and non-reproducible singleton features detected in only one biological sample. Following these filtering steps, 255 sample-associated metabolite features were retained for downstream analysis.”

We also added the NPClassifier reference to the reference list.

Scalable strategy: the authors claim in the discussion that their strategy is scalable: "Thus, while this study does not establish causal BGC-metabolite relationships, it provides a scalable prioritization strategy for selecting BGCs, regulators, growth stages, and metabolite features for downstream functional validation."

However, I miss the arguments/proof. Also, if it is so scalable as this sentence implies, how come the authors did not use/produce more omics data? The point needs some additional work to ensure that the lessons learnt from the current study will and can be used by others: is the strategy scalable by design? What makes it scalable? How come relatively little omics data was used in the current study? (this is a key thing to highlight as a lower number also impacts the chance of finding relevant links across omics layers) What are the least scalable parts and any mitigations or ideas on how to improve/accelerate these parts? Given the focus of the work on the prioritization framework, I think this should be worked out more. I value some of the other insights now mentioned in the discussion and I very much welcome the authors to also extend on those.

We agree with the reviewer that the term scalability is not accurate in the context used, and revised the Discussion to clarify that what was originally termed "scalability" refers to reduction of the candidate search space before validation, rather than to the volume of omics data generated in this study. Specifically, the paired-omics framework reduces the need to test every predicted BGC against every detected metabolite feature by using growth-stage-resolved transcriptomics to prioritize BGCs and regulators active under bioactive conditions, and metabolomics to prioritize features that accumulate alongside or after the onset of antifungal activity.

The revised manuscript text reads: " Nevertheless, this study establishes a prioritization strategy for natural product discovery by systemically reducing the candidate search space prior to functional validation. Rather than exhaustively testing every predicted BGC against all detected metabolite features, growth-stage-resolved transcriptomics enables identification of BGCs and regulatory elements that are specifically induced under bioactive conditions. In parallel, metabolomics independently prioritizes features that accumulate concurrently with, or subsequent to antifungal activity. This approach transforms global, unstructured comparison of BGCs,

metabolite features, and culture conditions, into a structured ranked set of candidates for targeted validation" on lines 729 – 737, and "Thus, while this study does not establish causal BGC-metabolite relationships, it demonstrates how paired transcriptomic and metabolomic profiling can prioritize candidate BGCs, regulators, growth stages, and metabolite features for downstream functional validation" on lines 744 - 746.

General comment: the line numbers in the response letter did not always correspond with the actual line numbers in the manuscript. My recommendation would be to always add the added manuscript sections to the response letter, so that it takes less time to track them in the manuscript.

We thank the reviewer for this helpful suggestion and apologize for any difficulty caused by mismatches between the response letter and manuscript line numbers. In the revised response letter, we have included the relevant revised manuscript text directly under each response, in addition to providing approximate line numbers where appropriate. This should make the changes easier to evaluate without requiring the reviewer to rely solely on line-number references.

Reviewer #2 (Comments for the Author):

The revised manuscript addresses most of the concerns raised in the previous rounds. There are, nevertheless, several parts where descriptions are either ambiguous or not completely supported by the data. These need to be corrected prior to publication.

L29. I think what's unknown is their products, not the functions.

This sentence was revised to "*Saccharomonospora xinjiangensis* XJ-54 is a rare actinomycete exhibiting antifungal activity against Fusarium phytopathogens, including *Fusarium oxysporum f. sp. cucumerinum* (FORC), and harbors numerous biosynthetic gene clusters (BGCs) whose products remain uncharacterized" in lines 27 -29.

L91-94. The papers cited here primarily discuss or apply integrating genomics and metabolomics. Transcriptomics is little taken account of if at all. Please cite a few articles where transcriptomics and metabolomics are integratively analysed or its need is more in depth discussed.

We agree that the original citations primarily supported genomics–metabolomics integration rather than integrated transcriptomic–metabolomic analysis. We have therefore added several references in which transcriptomics and metabolomics were jointly analyzed in the context of secondary metabolism. The revised text now reads: "Such transcriptome–metabolome integration has been used to link environmental or growth-phase cues to BGC activation and secondary metabolite production in Streptomyces, including studies of ethanol-induced metabolic remodeling, antifungal metabolite production during fermentation, and salt-induced secondary metabolism (19–21)" (lines 96–100). The following references were added to the revised manuscript:

19. Sekurova ON, Zehl M, Predl M, Hunyadi P, Rattei T, Zotchev SB. 2022. Targeted Metabolomics and High-Throughput RNA Sequencing-Based Transcriptomics Reveal Massive Changes in the Streptomyces venezuelae NRRL B-65442 Metabolism Caused by Ethanol Shock. Microbiol Spectr 10.

20. Gallart M, Dow L, Nowak V, Belt K, Sabburg R, Gardiner DM, Thatcher LF. 2025. Multi-omic investigation identifies key antifungal biochemistry during fermentation of a Streptomyces biological control agent. Microbiol Res 292:128032.

21. Otani H, Louie K, Faltane M, Lynde M, Bowen B, Mouncey NJ. 2026. Salt supplementation-induced metabolic reprogramming in Streptomyces coelicolor. mSystems 101128/msystems017 11:1–2.

L175. Convert the centrifugation unit from rpm to xg.

We converted the centrifugation speed from rpm to xg. The revised text now reports the centrifugation step as 1,500 × g for 10 min at 4°C in line 180.

L217-218. Describe what "alignments were processed" means.

We revised the Methods to specify the SAMtools processing steps. The revised text now states: "High-quality reads were aligned to the *S. xinjiangensis* XJ-54 reference genome using Bowtie2 (35), and resulting SAM alignment files were converted to BAM format, sorted, and indexed using SAMtools (36)." In lines 222 – 224.

L262. Although the parameters are provided in Zenodo, please describe some key parameters here such as the ppm and m/z tolerances, minimum peak height and peak width range. We revised the Methods to include the requested MZmine processing parameters, including m/z and ppm tolerances, minimum absolute peak height, and peak duration range. The revised text now reads: "MZmine parameters included m/z tolerances of 0.005 Da/20 ppm for chromatogram building, 0.0015 Da/3 ppm for isotope grouping, and 0.004 Da/8 ppm for feature alignment; the minimum absolute peak height was 1,000, and the peak duration range was 0-1.5 min. The full MZmine batch file, including all parameter settings, is available through Zenodo (https://doi.org/10.5281/zenodo.19368750)" in lines 267 – 271.

Figs 1C and 1E, and other similarly made figures. It is still unclear what each character means. Describe what each of them is in the figure legend. For example, what does "B" compare to in Fig 1C?
We revised the figure legends to clarify that letter annotations denote statistical groupings within each panel and that groups sharing at least one letter are not significantly different.

L361. Please provide the molar concentration of cycloheximide used.

We revised the figure legend to provide the molar concentration of cycloheximide. The concentration is now reported as 12.5 ppm, corresponding to 44.4 μM.

L377-378. This sentence is ambiguous. Was a subset of differentially expressed genes that had functional annotations used? Or were all the differentially expressed genes with functional annotations used? If the former is correct, please provide a justification for using only the subset. If the latter is the case, please rephrase this sentence.

We revised the sentence to clarify that all differentially expressed genes with KEGG or GO functional annotations were included in the KOBAS enrichment analysis. The revised text reads: "Functional enrichment analysis using KOBAS was performed on all differentially expressed genes assigned KEGG or GO functional annotations" in lines 384 – 385.

L416. Were z scores used for determining differentially expressed core biosynthetic genes instead of the fold changes? Please clarify which values were used and the criteria.

We revised the sentence to clarify that z-score normalization was used only to visualize BGC expression patterns. The revised sentence reads: "BGC expression patterns are shown as z-score-normalized mean TPM values of core biosynthetic genes, while genes classified as differentially expressed were identified using DESeq2, with significance defined as $\log_2$FC > 2, adjusted $P < 0.05$" in lines 421 - 424.

L419-420. TPM values themselves do not indicate whether a given is more highly transcribed than others. Rephrase this to accurately describe what is measured/compared.

We rephrased the sentence to avoid implying that TPM values alone establish that one BGC is more highly transcribed than another. The revised wording now describes only what was directly measured: normalized transcript abundance estimates for core biosynthetic genes, expressed as TPM values. We therefore refer to the "largest TPM values" rather than the "most highly transcribed clusters," while differential expression is described separately based on DESeq2 analysis. The revised text now reads: "Among the core biosynthetic genes analyzed, the largest TPM values were observed in Region 10 (ectoine), despite its core biosynthetic gene not being differentially expressed, and Region 1 (NRP-metallophore/NRPS), with peak values exceeding 60,000 TPM at day 7 and 4,000 TPM at day 3, respectively (Supplementary Table S2)" (lines 430–433).

L423-428. Please cite papers supporting these claims.

We added relevant citations to support our interpretations regarding the established role of ectoine as a compatible solute/osmoprotectant and the role of NRP-metallophore/siderophore systems in microbial iron acquisition. The citations are as follows:

56. Richter AA, Mais CN, Czech L, Geyer K, Hoeppner A, Smits SHJ, Erb TJ, Bange G, Bremer E. 2019. Biosynthesis of the Stress-Protectant and Chemical Chaperon Ectoine: Biochemistry of the Transaminase EctB. Front Microbiol 10:1–20.

57. Gräff ÁT, Barry SM. 2024. Siderophores as tools and treatments. npj Antimicrob Resist 2:1–17.

L443. BGCs do not encode metabolites. Rephrase it.

We rephrased the sentence to clarify that BGCs may contribute to antifungal metabolite production, rather than directly encode metabolites in lines 461 - 462.

Fig. S4. "Non-significant genes" sound as if they are irrelevant. Rephrase it.

We revised the Fig. S4 legend to replace “non-significant genes” with “genes that did not meet the differential-expression threshold.”

L483. It can be these regulators are irrelevant to the BGC expression. Since there's no evidence of multilayered architecture, state that this is a possibility, not the definitive mechanism.

We revised the text to avoid implying that the observed CSR expression patterns establish a definitive regulatory mechanism. The revised wording states that: “Within these individual BGCs, putative CSRs showed heterogeneous temporal expression patterns relative to core biosynthetic genes, ranging from synchronized to asynchronous profiles. Linking these regulators to BGC expression requires experimental validation.” in lines 490 – 493.

L485. What is shown is expression patterns and the relatively large number of TFs within the BGC, not regulatory architecture. Rephrase it to accurately indicate what the data and information show.

We revised the text to avoid referring to “regulatory architecture,”. The revised text now reads, “Among all BGCs, Region 6, a large (~168 kb) hybrid NRPS-PKS cluster, contained the greatest number of predicted regulatory genes and showed the most complex temporal expression patterns among both regulatory and core biosynthetic genes” in lines 498 – 500.

L511. There is an unnecessary dot.

The dot has been removed.

L577. Show quantities of this LC-MS feature over time from the time-series metabolomics data.

We examined the processed time-series metabolomics feature table for the m/z 257.04776 feature detected in fraction 19. Although this feature was reproducibly detected following chromatographic fractionation, including two additional independent fractionation steps, it was not retained in the unfractionated time-series feature table. Therefore, its abundance could not be quantified across the 3-, 5-, and 7-day samples. We have clarified this in the Results in lines 587 – 589.

L635. Again, TPM values do not indicate whether genes are significantly expressed. Change the tone of this claim. Also, prior studies do not claim that transcriptional inactivity is a universal feature.

We revised the statement to avoid implying that prior studies claim transcriptional inactivity is a universal feature of BGCs. For clarity, TPM values were used only for visualization, whereas differentially expressed genes were identified using DESeq2. The revised text now reads: "While it is often reported that many BGCs are transcriptionally silent under standard laboratory conditions (13), all 12 BGCs in *S. xinjiangensis* XJ-54 showed detectable transcription of core biosynthetic genes under the conditions tested, and 32 of 36 core biosynthetic genes were differentially expressed across growth stages based on DESeq2 analysis. These findings indicate that the standard cultivation conditions used here captured broad BGC transcriptional activity in this strain, and that time-resolved sampling revealed growth-stage-dependent BGC expression patterns that would be obscured by single-time-point analysis" in lines 632 – 638.

L650-656. Since there is no evidence that the analysed CSRs are indeed controlling expression of the BGCs, state that this is a possibility.

We revised the paragraph to clarify that the observed relationships reflect temporal expression patterns of putative CSRs and core biosynthetic genes, rather than direct evidence of regulatory control. The revised text now states: "These patterns are consistent with the possibility that CSRs contribute to temporal BGC expression, although direct regulatory roles require experimental validation" in lines 661– 663.

Re: mSystems00694-26R1 (**Deciphering temporal antifungal dynamics of a rare actinomycete via integrated omics**)

Dear Dr. Eddie Cytryn:

Thank you for the privilege of reviewing your work. Below you will find my comments, instructions from the mSystems editorial office, and the reviewer comments.

Please address the remaining minor reviewer comments.

1) providing the URL of the platform (https://pairedomicsplatform.bioinformatics.nl) and the project id (a7311d24-f581-4e5a-8d5c-558513dab7e7), in that case the interested reader will be able to track the current (and previous) version.

2) There are several parts where an extra space or punctuation is, which the authors should correct prior to publication.

Please return the manuscript within 60 days; if you cannot complete the modification within this time period, please contact me. If you do not wish to modify the manuscript and prefer to submit it to another journal, notify me immediately so that the manuscript may be formally withdrawn from consideration by mSystems.

Revision Guidelines
To submit your modified manuscript, log into the submission site at https://msystems.msubmit.net/cgi-bin/main.plex. Go to Author Tasks and click the appropriate manuscript title to begin. The information you entered when you first submitted the paper will be displayed; update this as necessary. Note the following requirements:

• Upload point-by-point responses to the issues raised by the reviewers in a file named "Response to Reviewers," NOT in your cover letter.
• Upload a compare copy of the manuscript (without figures) as a "Marked-Up Manuscript" file.
• Upload a clean .DOC/.DOCX version of the revised manuscript and remove the previous version.
• Each figure must be uploaded as a separate, editable, high-resolution file (TIFF or EPS preferred), and any multipanel figures must be assembled into one file.
• Any supplemental material intended for posting by ASM should be uploaded with their legends separate from the main manuscript. You can combine all supplemental material into one file (preferred) or split it into a maximum of 10 files with all associated legends included.

For complete guidelines on revision requirements, see our Submission and Review Process webpage. Submission of a paper that does not conform to guidelines may delay acceptance of your manuscript.

Data availability: ASM policy requires that data be available to the public upon online posting of the article, so please verify all links to sequence records, if present, and make sure that each number retrieves the full record of the data. If a new accession number is not linked or a link is broken, provide mSystems production staff with the correct URL for the record. If the accession numbers for new data are not publicly accessible before the expected online posting of the article, publication may be delayed; please contact production staff (mSystems@asmusa.org) immediately with the expected release date.

Publication Fees: For information on publication fees and which article types are subject to charges, visit our website. If your manuscript is accepted for publication and any fees apply, you will be contacted separately about payment during the production process; please follow the instructions in that e-mail. Arrangements for payment must be made before your article is published.

ASM Membership: Corresponding authors may join or renew ASM membership to obtain discounts on publication fees. Need to upgrade your membership level? Please contact Customer Service at Service@asmusa.org.

The ASM Journals program strives for constant improvement in our submission and publication process. Please tell us how we can improve your experience by taking this quick Author Survey.

Thank you for submitting your paper to mSystems.

Sincerely,
Trent Northen
Editor
mSystems

Reviewer #1 (Comments for the Author):

Thanks for considering my additional feedback.

I have checked the data identifiers and I could indeed locate MSV000101323 now.

Regarding the Paired Omics Data Platform submission - the current text reads that it is under review, but that is no longer the case it seems: it is available now, but the hard-coded URL does not work. I would refer to the the PoDP project by providing the URL of the platform (https://pairedomicsplatform.bioinformatics.nl) and the project id (a7311d24-f581-4e5a-8d5c-558513dab7e7), in that case the interested reader will be able to track the current (and previous) version.

Reviewer #2 (Comments for the Author):

I think the authors addressed all the comments and revised the manuscript accordingly. There are several parts where an extra space or punctuation is, which the authors should correct prior to publication.

Reviewer #1 (Comments for the Author):

Regarding the Paired Omics Data Platform submission - the current text reads that it is under review, but that is no longer the case it seems: it is available now, but the hard-coded URL does not work. I would refer to the the PoDP project by providing the URL of the platform (https://pairedomicsplatform.bioinformatics.nl) and the project id (a7311d24-f581-4e5a-8d5c-558513dab7e7), in that case the interested reader will be able to track the current (and previous) version.

We have included the approved project Id, and the revised text in lines 322 – 325 now reads: " The paired omics dataset has also been deposited in the Paired Omics Data Platform (55) (https://pairedomicsplatform.bioinformatics.nl) under project id; a7311d24-f581-4e5a-8d5c-558513dab7e7.2."

Reviewer #2 (Comments for the Author):

I think the authors addressed all the comments and revised the manuscript accordingly. There are several parts where an extra space or punctuation is, which the authors should correct prior to publication.

The manuscript has been revised throughout to remove extra spaces and correct punctuation errors.

Re: mSystems00694-26R2 (**Deciphering temporal antifungal dynamics of a rare actinomycete via integrated omics**)

Dear Dr. Eddie Cytryn:

Your manuscript has been accepted, and I am forwarding it to the ASM production staff for publication. Your paper will first be checked to make sure all elements meet the technical requirements. ASM staff will contact you if anything needs to be revised before copyediting and production can begin. Otherwise, you will be notified when your proofs are ready to be viewed.

Data Availability: ASM policy requires that data be available to the public upon online posting of the article, so please verify all links to sequence records, if present, and make sure that each number retrieves the full record of the data. If a new accession number is not linked or a link is broken, provide production staff with the correct URL for the record. If the accession numbers for new data are not publicly accessible before the expected online posting of the article, publication may be delayed; please contact ASM production staff immediately with the expected release date.

Publication Fees: For information on publication fees and which article types have charges, please visit our website. We have partnered with Copyright Clearance Center (CCC) to collect author charges. If fees apply to your paper, you will receive a message from no-reply@copyright.com with further instructions. For questions related to paying charges through RightsLink, please contact CCC at ASM_Support@copyright.com or toll free at +1-877-622-5543. CCC makes every attempt to respond to all emails within 24 hours.

ASM Membership: Corresponding authors may join or renew ASM membership to obtain discounts on publication fees. Need to upgrade your membership level? Please contact Customer Service at Service@asmusa.org.

Embargo Policy: A press release may be issued as soon as the manuscript is posted on the mSystems Latest Articles webpage. The corresponding author will receive an email with the subject line "ASM Journals Author Services Notification" when the article is available online.

Cover Image Submissions: If you would like to submit a potential Cover Image, please email a file and a short legend to msystems@asmusa.org. Please note that we can only consider images that (i) the authors created or own and (ii) have not been previously published. By submitting, you agree that the image can be used under the same terms as the published article. Image File requirements: TIF/EPS, 7.5 inches wide by 8.25 inches tall (at least 2,250 pixels wide by 2,475 pixels tall), minimum 300 dpi resolution (600 dpi preferred), RGB, and no figure elements, e.g., arrows or panel labels. The legend should be a short description of the image, 1-2 sentences recommended. Please use the ASM Journals Cover Image Sizing Tool (https://journals.asm.org/pb-assets/html/asm-cover-sizing-tool.html) to ensure that your proposed cover image meets our size requirements.

Thank you for submitting your paper to mSystems.

Sincerely,
Trent Northen
Editor
mSystems
