# Supplementary material for: Deciphering temporal antifungal dynamics of a rare actinomycete via integrated omics
Source: mSystems. 2026 Sep 28;11(10):e00694-26. doi: 10.1128/msystems.00694-26 (PMC13712795; doi:10.1128/msystems.00694-26)
Supplement: Supplementary file 2 [file msystems.00694-26-s0001.docx]

**SUPPLEMENTARY MATERIAL**

**Deciphering temporal antifungal dynamics of a rare actinomycete via integrated omics**

Hildah Amutuhaire^a, b^, Michael Dubovis^c^, Ivan Plyushchenko^c^, Judith Kraut-Cohen^a^, Tal Luzzatto-Knaan^c^, Jonathan Friedman^d^, Eddie Cytryn^a^ #

^a^	Institute of Soil, Water and Environmental Sciences, Agricultural Research Organization, Rishon LeZion, Israel.

^b^ Department of Plant Pathology and Microbiology, The Robert H. Smith Faculty of Agriculture, Food and Environment, The Hebrew University of Jerusalem, Rehovot, Israel.

^c^ The Interdisciplinary Center for Metabolomics, Department of Marine Biology, The Charney School of Marine Sciences, University of Haifa, Haifa, Israel.

^d^ The Institute of Environmental Sciences, The Hebrew University of Jerusalem, Rehovot, Israel.

#Address correspondence to Eddie Cytryn, eddie@volcani.agri.gov.il

**Figure S1.** Antifungal activity of methanol (MEOH) and acetonitrile (ACN) extracts against FORC. Antifungal activity was quantified as area under the growth curve (AUC), where lower values indicate stronger inhibition. TSB and cycloheximide (CHX, 12.5 ppm) served as negative and positive controls, respectively. Different letters indicate significant differences among treatments (one-way ANOVA followed by Tukey’s post hoc multiple-comparison test, *P* < 0.05). Data represent means ± SEM.

**Figure S2.** Global transcriptomic changes of *S. xinjiangensis* XJ54 across growth stages **(A)** Principal component analysis (PCA) based on log₂-transformed counts per million (CPM) gene expression values after filtering low-count genes (minimum row count = 10), of transcriptome profiles showing clear separation among samples collected at 3, 5, and 7 days. **(B)** K-means clustering of 2,410 differentially expressed genes (log₂FC ≥ 2, adjusted *P* < 0.05) revealing four distinct temporal expression patterns.

**Figure S3.** Pathway enrichment analysis of upregulated differentially expressed genes (DEGs) in *S. xinjiangensis* XJ54 identified by KOBAS 3.0 (n = 5). Bubble plots show significantly enriched pathways for three DEG expression clusters. , The x-axis indicates the input/background ratio, calculated as the number of cluster genes assigned to a pathway divided by the number of background genes assigned to that pathway. Bubble size represents the number of genes assigned to each pathway, and color denotes −log₁₀(p-value). (A) K1 cluster: pathways with progressively increased expression peaking at the stationary phase. (B) K2 cluster: pathways highly expressed during the logarithmic phase and lowest at the stationary phase. (C) K3 cluster: pathways associated with genes whose expression increased from day 3 to day 5 and then declined markedly by day 7. Cluster K4 was included in the enrichment analysis but is not shown because no pathways were significantly enriched.

**Figure S4.** Temporal expression dynamics of BGC biosynthetic and regulatory genes in *S. xinjiangensis* XJ54 across growth stages. Line plots show Z-score–normalized expression of biosynthetic (red) and regulatory (green) genes within individual BGC regions at days 3, 5, and 7. Solid lines indicate significantly differentially expressed genes (DEGs; *P* < 0.05, |log₂FC| > 2), while dashed lines denote non-significant genes. Error bars represent the standard error of the mean (SEM) of TPM values across 5 replicates.

**Figure S5.** Antifungal activity of *S. xinjiangensis* stationary phase CFS chromatographic fractions against FORC. Area under the curve (AUC) of FORC growth in response to fractions 1–13 (A) and 14–19 (B) in 96-well plate assays; lower AUC values indicate greater FORC growth inhibition. Fractions 1-13 and 14 -19 were tested and plotted as separate assay sets; therefore, AUC values and statistical groupings should be interpreted within each panel rather than compared directly between panels. Controls: PDB medium (negative), 1% DMSO (solvent), and 12.5 ppm cycloheximide (CHX, positive). Different letters denote significant differences among treatments (Kruskal-Wallis test, *P* < 0.05). Groups sharing at least one letter are not significantly different, whereas groups with no letters in common are significantly different. Data are mean ± SEM (n = 4 for A; n = 5 for B).

**Figure S6.** Bace Peak Chromatogram of fraction 19 overlaid with blank in negative mode. Sample in Blue, Blank in Black.

**Figure S7.** Bace Peak Chromatogram of fraction 19 overlaid with blank in positive mode. Sample in Blue, Blank in Black.

**Figure S8.** MS1 (left) and MS2 (right) spectra of main peak of fraction 19.

**Figure S9.** Mirror plot of the experimental (blue, top) and theoretical (red, bottom) isotopic pattern for the molecular formula C_14_H_9_ClN_2_O. Mass adduct [M+H]^+^. Formula was predicted in [BUDDY](https://www.nature.com/articles/s41592-023-01850-x) tool based on isotopic pattern. Theoretical isotopic pattern for C_14_H_9_ClN_2_O was computed by [envipat](https://pubs.acs.org/doi/full/10.1021/acs.analchem.5b00941).
